# Mental health literacy about depression: a survey of portuguese youth

**DOI:** 10.1186/1471-244X-13-129

**Published:** 2013-05-07

**Authors:** Luís M Loureiro, Anthony F Jorm, Aida C Mendes, José C Santos, Ricardo O Ferreira, Ana T Pedreiro

**Affiliations:** 1 Health Sciences Research Unit – Nursing, Mental Health and Psychiatry Nursing, Nursing School of Coimbra, Avenida Bissaya Barreto, Apartado 7001, Coimbra 3046-851, Portugal; 2 Melbourne School of Population Health, University of Melbourne, Melbourne, Australia; 3 Health Sciences Research Unit – Nursing, Nursing School of Coimbra, Coimbra, Portugal

**Keywords:** Mental health literacy, Depression, Youth, Help-seeking

## Abstract

**Background:**

Depression is a common disorder in adolescents and young adults, but help seeking is low. Mental health literacy about depression is a key concept to plan interventions for improving help seeking. This study aimed to evaluate youth mental literacy about depression in order to design school-based interventions.

**Methods:**

During 2012, a survey was conducted with a stratified cluster sample of 4938 Portuguese young people between 14 and 24 years of age. Following the presentation of a vignette describing depression, a series of questions was asked concerning: recognition of the disorder; knowledge of professional help and treatments available; knowledge of effective self-help strategies; knowledge and skills to give first aid and support to others; and knowledge of how to prevent this disorder.

**Results:**

In response to an open-ended question, around a quarter of the participants failed to recognize depression in the vignette. When asked about the potential helpfulness of various people, most of the participants considered mental health professionals, family and friends to be helpful. However, teachers, social workers and a helpline were less likely to be considered as helpful. With regard to medications, vitamins received more positive views than psychotropics. Some interventions were frequently rated as likely to be helpful, whereas for others there was a lack of knowledge about their effectiveness. A positive finding is that alcohol and tobacco consumption were seen as harmful. When asked about mental health first aid strategies, participants supported the value of listening to the person in the vignette and advising professional help, but some unhelpful strategies were commonly endorsed as well.

**Conclusion:**

Deficits were found in some aspects of depression literacy in Portuguese youth. Therefore intervention in this area is needed.

## Background

Mental health literacy has been defined as “knowledge and beliefs about mental disorders which aid their recogntion, management or prevention”. It involves knowledge which allows a person to take action to improve their own mental health or that of others [1]. Mental health literacy includes a set of interconnected components which are: the ability to recognize disorders in order to facilitate help seeking; knowledge of professional help and treatments available; knowledge of effective self-help strategies; knowledge and skills to give first aid and support to others; and knowledge of how to prevent mental disorders [2].

Mental health literacy is particularly important during adolescence and early adulthood. This is the peak period for the onset of mental disorders. Half of the people who will suffer from a mental disorder have their first episode before 18 years of age [3]. Furthermore, this period of life is characterized by significant changes and transitions, which means that mental disorders can have a profound impact on a young person’s personal, social, occupational, physical and emotional life, and serious long-term repercussions for their personal and professional future [1,2,4].

While the prevalence of mental disorders in young people is high, ranging between 15 and 20%, they have a low rate of contact with health care [4,5]. Many young people do not seek help or postpone help seeking due to various personal and structural barriers such as: fear of stigma and discrimination associated with depression (e.g. it is seen as a weakness); concerns about confidentiality and anonymity; lack of knowledge about the availability of help; the widespread perception that, within this age group, the signs and symptoms reflect only a temporary age crisis; and lack of appropriate responses from both peers and adults, often because they do not know how to act, allowing the problem to get worse [6,7]. Many of these barriers relate to limited mental health literacy. The consequent lack of help seeking or delay in help seeking may lead to worse health outcomes and possibly to chronicity [8].

While mental health literacy of young people has been researched in some English-speaking countries [9-11], there are limited data from the rest of the world, including Portugal. Such studies are needed in order to plan interventions to improve recognition, help-seeking, prevention and support provided by others. Within the mental health literacy area, studies on the range of mental disorders affecting young people are necessary. However, this study focusses on depression, which is one of the most important mental disorders in young people. By the age of 18, one in five has experienced depression [4], and the prevalence of this disorder in youth is between 4 and 8% [12,13].

The aim of the current study was therefore to investigate depression literacy in a sample of the Portuguese youth. We specifically sought to evaluate the different components of the concept of mental health literacy relevant to depression: a) recognition of depression; b) knowledge of professional help and treatments available; c) knowledge of effective self-help strategies; d) knowledge and skills to give first aid and support to others; and e) knowledge of how to prevent depression.

## Methods

### Sample

This study is based on a questionnaire in the form of a written self-report, administered to a representative sample of 4938 adolescents and young adults, 43.3% males and 56.7% females, aged between 14 and 24 years (mean age of 16.75 years and standard deviation of 1.62 years). They were residing in the central region of mainland Portugal in the districts of Coimbra, Viseu, Leiria, Aveiro, Guarda and Castelo Branco. They were attending 3rd cycle of primary and secondary schools (7th to 12th grade), which are integrated into the Regional Direction of Education – Centre (DREC), from the NUTS III - Centre of Territorial Statistics Units of Portugal.

The sample size was calculated from the population of resident young people, according to the data statistics of residents from the NUTS III - Centre (Territorial Statistics Units of Portugal, in 2010 the region covered by the study [14]). Stratification was accomplished according to the population representativeness in each region of NUTS III - Centre, taking into consideration the desired sample size (n = 5000), with a sample size error of 1.3%. The sample selection was based on four strata (NUTS III region; Rural/Urban; Coast/Interior; education degrees).

The sampling technique used was the multi-stage clustered-stratified method. From the list of all 271 management units in the DREC, 50 schools were randomly selected using the Research Randomizer Software, while the strata were met. From each school selected, 3 or 4 classes were randomly selected (using the software mentioned above) and, from each class, all students were selected.

### The questionnaire

To assess the mental health literacy of adolescents and young people about depression, we used the Questionnaire for Assessment of Mental Health Literacy - QuALiSMental, after it had been translated, adapted and validated for the Portuguese population. Detailed information about the questionnaire can be found in a publication on the matter [15].

This questionnaire is composed of different sections, each one consisting of several questions concerning the evaluation of a component of mental health literacy. The questionnaire begins with instructions for completion and socio-demographic questions (gender, age, residence, district and parents’ qualifications). Then there is a vignette (Case vignette used in the questionnaire) describing a case of depression in a 16-year-old girl named Joana, which serves as the target for all the questions in the following sections. The case satisfies the diagnostic criteria for Major Depressive Episode in DSM-IV-TR [16].

### Case vignette used in the questionnaire

Joana is a 16 years old girl who is feeling unusually sad for the last few weeks. She feels tired all the time and she has problems to fall asleep and when she can fall asleep she wakes up many times. She has lost appetite and lately has lost some weight. It is difficult for her to concentrate and her grades have dropped. Even the daily activities seem a lot to her, which made her postpone some decisions. Her parents and friends are very worried about her.

To assess *recognition of depression* from the vignette presented, the following question was asked: “In your opinion, what is going on with Joana?”. The response format was multiple choice and the possible answers were: “I do not know”, “There is nothing wrong with her”, “She has a problem”, “Depression”, “Schizophrenia”, “Psychosis”, “Mental Illness”, “Bulimia, “Stress, “Nervous Breakdown”, "Substance Abuse (e.g.: alcohol)”, “age crisis”, “Psychological/Mental/Emotional Problems”, “Anorexia”, “Alcoholism”, “Cancer” and “Other (specify which)”.

An answer was considered correct if the respondent chose depression, either alone or in combination with one of the following options: has a problem, mental illness, stress, psychological/mental/emotional problems.

To assess *knowledge of professional help and treatments available*, the respondents were asked: “There are different people and health professionals who can help Joana”, respectively: General Practitioner; teacher; psychologist; nurse; social worker; psychiatrist; telephone helpline; close family member; and close friend. For each item, participants could check one of the following three response options: helpful, harmful and neither or do not know. Concerning treatments/products available, the following were presented: vitamins, tea, tranquillizers, antidepressants, antipsychotics and sleeping pills. Participants should choose from the three options: helpful, harmful and neither or do not know.

To assess *knowledge of interventions*, the following statement was presented: “There are different activities that could help Joana. Point out for each of them your opinion”. The following list was then presented: Becoming more physically active; Getting relaxation training; Practicing meditation; Getting acupuncture; Getting up early each morning and getting out in the sunlight; Receiving therapy with a specialized professional; Looking up a website giving information about her problem; Reading a self-help book on her problem; Joining a support group of people with similar problems; Going to a specialized mental health service; Using alcohol to relax; Smoking cigarettes to relax. For each option, the participants had to mark one of the following three choices: helpful, harmful and neither or don’t know.

To assess *knowledge and skills to give first aid and support to others*, the following actions were presented: Listen to her problems in an understanding way; Talk to her firmly about getting her act together; Suggest she seek professional help; Make an appointment for her to see a GP with her knowledge; Ask her whether she is feeling suicidal; Suggest she have a few drinks to forget her troubles; Rally friends to cheer her up; Not acknowledge her problem, ignoring her until she gets over it; Keep her busy to keep her mind off problems; Encourage her to become more physically active. The response options were: helpful, harmful and neither or don’t know.

Finally, to assess *knowledge of how to prevent mental disorders*, the following items were presented: Keeping physically active; Avoiding situations that might be stressful; Keeping regular contact with friends; Keeping regular contact with family; Not using drugs; Never drinking alcohol; Making regular time for relaxing activities; Having a religious or spiritual belief. The response options were *yes*, *no* and *I don*’*t know*.

The questionnaire was administered to participants in the classroom setting. The response time for the questionnaire was 40–45 min.

### Ethical approval

The study and the survey questionnaire were approved by the General Directorate for Innovation and Curriculum Development of the Ministry of Education of the Portuguese Government and the Committee of Ethics of UICISA-E of ESEnfC. Given the characteristics of the sample (mostly minors), the questionnaire was accompanied by a consent form to be signed by parents/guardians or, in cases where the youths were aged 18 years or over, a consent form on their own behalf.

### Statistical analysis

Data were analyzed using the IBM-SPSS 20.0 software. As this is a descriptive exploratory study, we calculated the appropriated summary statistics and the absolute and percentage frequencies in order to meet the objectives of the study, using the procedure Multiple Response Table. We also calculated the 95% confidence interval for the percentage giving a correct response to the question on the recognition of depression.

## Results

### Recognition of depression

Table 1 shows the responses given by participants to the question “*In your opinion*, *what is going on with Joana*?”. Depression was the most common answer (61.1%), followed by stress (47.3%), psychological/mental/emotional problems (40.8%), nervous breakdown (33.8%) and anorexia (16.4%).

**Table 1:** Percentage of respondents endorsing each category to describe the problem shown in the vignette describing a case of depression (N = 4938)

	**%**
Depression	67.1
Stress	47.3
Psychological/mental/emotional problems	40.8
Nervous breakdown	33.8
Has a problem	22.7
Anorexia	16.4
Age crisis	14.8
Bulimia	6.5
Mental illness	5.5
Substance abuse	3.2
I don’t know	2.7
Cancer	1.2
Alcoholism	.9
Psychosis	.9
Schizophrenia	.7
Nothing	.4

From the three options concerning Joana’s situation, *I don*’*t know*; *nothing* and *has a problem*, it was found that 22.7% stated that Joana has a problem (they could associate a disorder from the existing list), 2.7% reported that they did not know what it is and 4% reported that there is nothing wrong with her. Only 5.5% of participants stated that Joana’s problem was a mental illness, while 14.8% considered that it was an age crisis.

The corrected categories were grouped to allow calculation of the percentage recognizing depression. It was found that 27.2% of the participants (95% CI: 26.0-28.5) recognized Joana’s situation as depression.

An analysis performed a posteriori on the combinations of items that reflect a correct identification of the vignette revealed that, of the 27.2% of participants who recognized the signs and symptoms described as indicating depression, 12.0% marked only depression while the remaining 15.2% associated depression with other options considered valid (mental illness; stress; psychological problems) (Table 1).

### Knowledge of professional help and treatments available

Concerning the different people, including professionals, who could help Joana, Table 2 shows that the health professionals most often considered likely to be helpful were psychologist (89.0%), general practitioner (74.6%), psychiatrist (55.1%) and nurse (49.1%). Informal supportive persons, such as friends (80.9%) or family members (75.1%), were very frequently rated as helpful, exceeding all of the professionals except for psychologists.

**Table 2:** Percentage of respondents endorsing various potential types of help (N = 4938)

	**Helpful**	**Harmful**	**Neither or don’t know**
***Different people who could possibly help***			
A family doctor	74.6	2.1	23.2
A teacher	27.6	8.7	63.7
A psychologist	89.0	2.3	8.7
A nurse	49.1	4.1	46.8
A social worker	14.6	16.4	69.0
A psychiatrist	55.1	9.5	35.5
A telephonic helpline	16.4	23.2	60.5
A close family member	75.1	3.3	21.6
A close friend	80.9	2.5	16.6
***Medicines***			
Vitamins	65.5	4.2	30.3
Tea	54.0	3.6	42.3
Tranquillizers	29.9	30.4	39.7
Antidepressants	37.7	28.6	34.1
Antipsychotics	8.8	37.3	53.9
Sleeping pills	29.3	30.9	39.8
***Interventions***			
Becoming more physically active	66.2	5.2	28.6
Getting relaxation training	82.9	1.2	15.8
Practicing meditation	62.7	2.9	34.4
Getting acupuncture	23.4	7.4	69.3
Getting up early each morning and getting out in the sunlight	27.6	9.8	62.6
Receiving therapy with a specialized professional	74.8	2.6	22.5
Looking up a web site giving information about her problem	45.9	12.7	41.4
Reading a self-help book on her problem	48.3	8.3	43.4
Joining a support group of people with similar problems	49.9	9.8	40.3
Going to a specialized mental health service	59.3	7.2	33.5
Using alcohol to relax	2.8	84.8	12.4
Smoking cigarettes to relax	4.0	83.6	12.4
***Knowledge and skills to give first aid and support to others***			
Listen to her problems in an understanding way	95.2	.7	4.1
Talk to her firmly about getting her act together	48.6	14.3	37.1
Suggest she seek professional help	77.2	4.6	18.2
Make an appointment for her to see a GP with her knowledge	60.8	6.5	32.7
Ask her whether she is feeling suicidal	16.6	52.0	31.8
Suggest she have a few drinks to forget her troubles	3.6	87.1	12.9
Rally friends to cheer her up	73.8	4.5	21.7
Not acknowledging her problem, ignoring her until she gets over it	3.1	84.5	12.4
Keep her busy to keep her mind off problems	62.7	8.5	28.8
Encourage her to become more physically active	48.5	5.8	45.7

The data in Table 2 also show that the vast majority of young people respond either “don’t know” or “neither helpful nor harmful” for teachers (63.7%), social workers (69.0%) and the telephone helpline (60.5%). The source of help most frequently considered harmful was the telephone helpline (23.2%).

With regard to medicines and products, the ones most frequently rated as helpful were vitamins (65.5%) and tea (54.0%), followed by antidepressants (37.7%) and tranquilizers (29.3%). The medications most frequently considered harmful were antipsychotics (37.3%), hypnotics (30.9%), tranquilizers (30.4%) and even antidepressants (28.6%). The percentage of participants who responded “don’t know” or “neither” was highest in the case of antipsychotics (53.9%), but it was also substantial for teas (30.3%) and vitamins (30.3%).

### Knowledge of effective interventions

When asked about the effectiveness of interventions (Table 2), participants identified as the most helpful ones: *getting relaxation training* (82.9%), *receiving therapy with a specialized professional* (74.8%), *becoming more physically active* (66.2%), *practicing meditation* (62.7%) and *going to a specialized mental health service* (59.3%). Approximately half the sample considered helpful *joining a support group of people with similar problems* (49.9%), *reading a self*-*help book on her problem* (48.3%) and *looking up a web site giving information about her problem* (45.9%). There was low endorsement of *using alcohol to relax* (2.8%) and *smoking cigarettes to relax* (4.0%). Moreover most of the respondents considered these harmful (84.8% and 83.6%).

However, there were a number of interventions which had more than half the sample responding that they either did not know or considered them neither helpful nor harmful. This was true for: *getting acupuncture* (69.3%) and *getting up early each morning and getting out in the sunlight* (62.6%). Such responses were also common for *looking up a web site giving information about her problem* (41.4%), *reading a self*-*help book on her problem* (43.4%) and *joining a support group of people with similar problems* (40.3%).

### Knowledge and skills to give first aid and support to others

To evaluate this component, a list of behaviors that young people might have towards Joana was provided (Table 2). The behaviors that respondents most frequently considered useful were *listen to her problems in an understanding way*, which was endorsed by 95.2% of the participants; *suggest she seek professional help* (77.2%); *rally friends to cheer her up* (73.8%) and *make an appointment for her to see a GP with her knowledge* (60.8%).

Approximately half of the sample also considered it useful to *talk to her firmly about getting her act together* (48.6%) and *encourage her to become more physically active* (48.5%).

It should be noted that a considerable percentage (over 80.0%) considered it harmful to *suggest she have a few drinks to forget her troubles* (87.5%) and *not acknowledge her problem*, *ignoring her until she gets over it* (84.5%).

Concerning asking Joana if she has suicidal thoughts (*ask her whether she is feeling suicidal*), most Portuguese young people considered this to be harmful (52.0%) or did not know of its usefulness (31.8%). There was also low endorsement of *firmly talk to her about getting her act together* (37.1%).

### Knowledge of how to prevent mental disorders

To assess beliefs concerning the prevention of depression, the following question was asked: “*do you think the risk of developing a condition like Joana*’*s would reduce if young people*…?”. As shown in Table 3, the majority of participants felt that the activities presented reduced the risk of suffering from a problem like Joana’s, except for the item *having a religious or spiritual belief* (17.5%). In this case, 38.9% said it would have no impact and 43.6% did not know.

**Table 3:** Percentage of respondents endorsing each item on beliefs about prevention (N = 4938)

**Beliefs about prevention**	**Yes**	**No**	**I don’t know**
Keeping physically active	64.0	12.4	23.6
Avoiding situations that might be stressful	83.6	6.8	9.5
Keeping regular contact with friends	85.6	4.9	9.5
Keeping regular contact with family	86.2	4.8	9.0
Not using drugs	82.0	8.5	9.5
Never drinking alcohol	74.3	12.5	13.2
Making regular time for relaxing activities	72.0	6.6	21.5
Having a religious or spiritual belief	17.5	38.9	43.6

It is also noteworthy that 23.6% of the participants did not know whether the risk of developing depression would decrease if they maintain regular physical activity and 21.5% for *making regular time for relaxing activities*.

## Discussion

Recognition of depression is a factor that can facilitate professional help seeking [17] and consequently reduce the prolonged suffering, suicide risk and developmental consequences of an untreated mental disorder [18]. Given the potential importance of correct recognition, the percentage of participants in this study who were able to correctly label the person in a vignette as experiencing depression was lower than desirable, even though we allowed responses where the label of depression was used together with other labels that might facilitate appropriate help seeking, such as stress, psychological/mental/emotional problems and mental illness. Correct recognition was lower in this study than in some others which have used a similar methodology [1]. Some insight into the factors behind this low level of recognition can be gained by examining the alternative labels that were commonly used. First, there was a high frequency of using the label “nervous breakdown”. This is a nonspecific term, which is generally used to characterize almost all changes in mental health and does not specify the particular problem. Secondly, around 15% of young people associated the signs and symptoms with a temporary age crisis. This characterization of a depressed teenager as being in a temporary age crisis, which is a common view in society of what constitutes normal adolescence, can mean a lack of appreciation of the suffering seen by family and friends and become a barrier to help seeking or even to the provision of aid.

Other participants associated the situation described in the vignette with “anorexia”, which clearly demonstrates an inability to take acccount of the full range of signs and symptoms, overvaluing the sign of weight loss and the symptom of loss of appetite, and possibly leading to inappropriate help seeking.

While the inability of many young people to recognize depression may have implications for help-seeking, most did believe in the usefulness of the psychologists and general practitioners and, at a similar level, in informal sources such as friends and close family, which indicates an understanding of the need for intervention by another person, professional or not, and the difficulties experienced by the person facing these symptoms. According to the Portuguese clinical practice recommendations [19], primary health care should be the first place to go when young people experience the first symptoms of depression, and they should have an appointment with a general practitioner. In cases of severe depression, it is recommended that the GP refers the adolescent or young adult to specialized psychiatric services.

Many young people showed a lack of knowledge of the potential usefulness of social workers and teachers. In the case of social workers, this may be due to a perception that this profession acts only at the level of basic solutions, such as providing economic and social resources for problematic situations. As for teachers, the issues of anonymity and confidentiality may arise, and particularly a perception that the teacher’s knowledge of the young person’s situation may have direct implications for their academic success. In this sense, the reluctance to seek help from teachers may seem to be in the interests of the young.

The fact that nurses are also considered useful, although less so than other formal sources (psychologist, GP and psychiatrist), highlights their proximity to adolescents in terms of health interventions in schools.

Telephone helplines are a readily available way to ask for help, but most young people either lacked knowledge of their potential helpfulness or were suspicious as to their usefulness. One possible reason for this finding may be that, when young people are seeking help,it is fundamental that the helper be trustworthy and maintain confidentiality and this is difficult to guarantee when the person is not known to them [5].

As far as medications and therapeutic products are concerned, we found that non-prescription products such as vitamins and teas, which have no supporting evidence, are believed by many young people to be useful, although some are more sceptical or uncertain. However, as in other studies [1], psychiatric drugs are mostly viewed as harmful or young people are uncertain as to their usefulness, which to some extent may influence non-adherence to treatment when prescribed by a healthcare professional. In Portugal, medication is recommended for specific cases, in severe depressions, along with other interventions, like psychotherapy and intervention with the family [19].

Considering beliefs about the effectiveness of self-help strategies, we can see two aspects in the responses. Some young people believe in the usefulness of self-help strategies, such as relaxation training, physical exercise, consulting a website, and reading a self-help book about the problem, which is to some extent consistent with the emphasis given to the use of vitamins and tea for treatment of depression. On the other hand, other young people doubt or do not even know about the usefulness of these strategies, which may to a certain extent be due to the reduced investment made in the promotion of health behaviors. Alcohol and tobacco consumption are exceptions. Young people clearly believe that these substances aggravate existing mental health problems.

Some self-help strategies, like exercise, relaxation and self-help books, have evidence of effectiveness for depression, so it is of concern that only a substantial minority of respondents endorsed the potential helpfulness of such strategies. Given such beliefs, young people may not adopt potentially useful strategies, which may help in mild levels of depressive symptoms, particularly if adopted under the supervision of health professionals. These findings indicate the need for public awareness campaigns on mental health that make clear reference to potentially useful self-help strategies, as previously proposed [20].

In terms of knowledge and skills to give first aid and support to others, there is evidence that when people experience a mental health problem like depression, they are more likely to seek help when it is suggested by someone close to them, whether it is a friend or a family member [21]. It was encouraging to see that young people who participated in this study showed a marked predisposition to listen to the person who needs help and then suggest professional help-seeking or even make an appointment for her to see a GP with her knowledge, consistent with the guidelines of programs like Mental Health First Aid [3]. However, it is worrying that, as found in other studies [22], a considerable percentage of young people believed that it is not useful, and indeed harmful, to ask the person who is experiencing depression if she/he has suicidal thoughts. The reluctance to ask about suidical thoughts may be explained by the belief that just asking the person about such thoughts may put the idea in her/his head.

The strategy of keeping the person busy to keep her mind off problems was considered useful by the vast majority of young people, and a majority considered it useful to talk to her firmly about getting her act together. It is possible that distraction leads a person to temporarily forget their problems. However, this is not conducive to seeking help or treatment. As for talking firmly, firmness and even criticism of the person with mental illness is not beneficial, and in many cases is counterproductive, as it leads the person to isolation and not sharing problems and may increase their distress. Mental health professionals generally do not see either of these strategies as likely to be helpful [22].

Concerning the last component of mental health literacy, knowledge of how to prevent mental disorders, adolescents and young people have a clear belief that is possible to prevent mental illness [23], specifically by avoiding situations that might be stressful, promoting activities such as regular contact with friends and family, and not using drugs. Mental health professionals also endorse the maintenance of regular contact with friends and family, and not using drugs and alcohol, as preventive strategies for mental disorders [23]. However, they consider that avoiding stressful situations could be harmful for young people, leading to the development of anxiety disorders [23].

Concerning physical activity, the findings were consistent for its use as a self-help strategy as well as for prevention, with a significant percentage not knowing whether it is likely to be helpful in both instances. This is of concern, because mental health professionals also recommend physical activity as a strategy for preventing depression [23].

At the same time, the religious and spiritual beliefs that are presented in the literature as a protective mechanism against suicidal thoughts were not seen as preventive by the majority of young people in this study (82.5%).

## Conclusion

The results from this descriptive study suggest the need to develop programs aimed at increasing all the components of mental health literacy. Adolescence and youth are very important periods for acquiring knowledge and adopting behaviors that are transferred into adult life.

Different programs for youth, like the Portuguese webprogram “Feliz Mente” (http://felizmente.esenfc.pt), should be widely disseminated in schools in order to increase mental health literacy in young people. Schools are important settings for such intervention programs, because it is where teenagers and young people spend most of their time.

These programs should also target adults who work with adolescents and young people. An example is the program Youth Mental Health First Aid [1,3] which trains parents, teachers, school workers and primary care nurses in how to assist adolescents developing a mental health problem or in a mental health crisis situation. Education professionals need to be empowered to act, because they are important persons in students’ lives. They spend much time with young people and should therefore be able to recognize the first signs and symptoms and give first aid and support.

By increasing mental health literacy about depression, it is hoped to increase help-seeking behaviors, facilitate first aid given to others and reduce the delay between the first signs and symptoms and seeking help from a professional.

## Competing interests

The authors declare they have no competing interests.

## Authors’ contributions

LML and ATP wrote the manuscript. LML coded all the responses and performed the analyses. LML secured funding for the survey and co-designed the survey, co-developed the coding scheme, co-developed the scoring for the sample responses and approved this manuscript. AFJ co-designed the survey, co-developed the coding scheme and co-developed the scoring for the sample responses. ACM, JCS, ROF and ATP co-designed the survey and approved the manuscript. AFJ approved the manuscript. All authors read and approved the final manuscript.

## Pre-publication history

The pre-publication history for this paper can be accessed here:

http://www.biomedcentral.com/1471-244X/13/129/prepub
